# An Event Related Potentials Study of the Effects of Age, Load and Maintenance Duration on Working Memory Recognition

**DOI:** 10.1371/journal.pone.0143117

**Published:** 2015-11-16

**Authors:** Diego Pinal, Montserrat Zurrón, Fernando Díaz

**Affiliations:** Applied Cognitive Neuroscience Laboratory, Department of Clinical Psychology and Psychobiology, Faculty of Psychology, Universidade de Santiago de Compostela, Galiza, Spain; Banner Alzheimer's Institute, UNITED STATES

## Abstract

Age-related decline in cognitive capacities has been attributed to a generalized slowing of processing speed and a reduction in working memory (WM) capacity. Nevertheless, it is unclear how age affects visuospatial WM recognition and its underlying brain electrical activity. Whether age modulates the effects of memory load or information maintenance duration, which determine the limits of WM, remains also elusive. In this exploratory study, performance in a delayed match to sample task declined with age, particularly in conditions with high memory load. Event related potentials analysis revealed longer N2 and P300 latencies in old than in young adults during WM recognition, which may reflect slowing of stimulus evaluation and classification processes, respectively. Although there were no differences between groups in N2 or P300 amplitudes, the latter was more homogeneously distributed in old than in young adults, which may indicate an age-related increased reliance in frontal vs parietal resources during WM recognition. This was further supported by an age-related reduced posterior cingulate activation and increased superior frontal gyrus activation revealed through standardized low resolution electromagnetic tomography. Memory load and maintenance duration effects on brain activity were similar in both age groups. These behavioral and electrophysiological results add evidence in support of age-related decline in WM recognition theories, with a slowing of processing speed that may be limited to stimulus evaluation and categorization processes -with no effects on perceptual processes- and a posterior to anterior shift in the recruitment of neural resources.

## Introduction

As humans age, there is a certain generalized decline in cognitive capacities [1]. Recognition processes, defined as the identification of items (people, objects, words, etc.) as having been previously encountered or experienced, are assumed to be among those abilities affected by age-related decline in cognitive performance [2,3]. However, most research has focused on how aging affects long-term episodic memory recognition, while little is known about such effects on working memory (WM) recognition.

WM is a capacity-limited system that comprises the ability to mentally manipulate and hold in mind for brief periods of time (i.e. a few seconds) small amounts of information that are no longer available in the environment [4,5]. The limits of WM capacity seem to be determined by the amount and complexity of information to be encoded into memory, the so-called memory load [6,7], and by the amount of time it has to be held in mind [8]. Interestingly, some authors have suggested that the limits of WM capacity may be reduced in old adults relative to young adults [9,10]. Also, it has been hypothesized that age-related decline in this and other cognitive capacities is related to a generalized slowing of processing speed [11] and to a decrease in processing resources [12].

These age-related changes are supposed to stem from differences in brain activity between young and old adults. Therefore, the registration and analysis of EEG activity during delayed match to sample (DMS) and Sternberg tasks may represent an optimal means of testing for age-related differences in the brain electrical activity underlying WM recognition processes. First, the aforementioned tasks enable the study of recognition processes in isolation from encoding and/or maintenance processes [13]. Also, they enable experimental manipulation of memory load and of the time that information has to be held in mind (maintenance period), a fact that facilitates the testing of WM capacity. Second, use of the event related potentials (ERPs) technique enables the study of brain electrical activity in response to a defined event (e.g. presentation of a stimulus), with a temporal precision of milliseconds.

As regards the generalized slowing of processing speed, research undertaken using the aforementioned tasks in combination with the ERP technique has shown longer N1 [14,15] as well as P300 [14,16,17] latencies in old than in young adults. Although this pattern of results is generally consistent with a slower processing speed with aging, the effects of healthy aging on the latency of P2 and N2 components remains controversial, since mixed results have been obtained during WM recognition in previous studies [15,16]. Likewise, regarding the reduction in the capacity to allocate processing resources, previous research revealed larger P1 amplitudes [14] and lower P300 amplitudes [14,16,17] in old than in young adults, pointing to age-related differences in the allocation of processing resources. Nevertheless, mixed results have been obtained during WM recognition for P2 and N2 components amplitude [15,16]. With respect to these two components, differences in task difficulty (9 possible locations vs 50 locations) between studies may underlie the discrepancy of previous results. Consequently, it is of great interest to study whether age-related effects in these components interact with memory demands in order to shed light on this issue.

A third hypothesis assumes that old adults may have a reduced WM capacity relative to young adults. Hence, it has been suggested that old adults show similar modulations in brain activity as young adults, but when facing lower levels of memory demand [18,19]. Previous research has revealed lower N1 [20,21] and P300 [20–22] amplitudes for high than for low memory load conditions in young participants during WM recognition in DMS tasks; and, albeit similar results have also been observed with older adults, no significant interaction between WM load and age-related effects on brain electrical activity was observed [14,17]. These latter studies used visual presentation of 1, 3 or 5 digits, and, therefore, it is still unclear whether the hypothesized higher sensitivity of older adults to memory load would be observed for more complex materials and different means of manipulating WM load.

In addition, during the recognition stage of DMS tasks, the amount of time that information has to be actively maintained in WM has been associated with larger N2 amplitudes after long than after short maintenance periods in young adults [22,23]. However, to the best of our knowledge, no previous ERP studies have assessed the effects of the maintenance period duration in old adults at the recognition stage of a DMS task. Nevertheless, as this is an aspect that set the limit of WM capacity, the existence of age-related differences in brain electrical activity during WM recognition due to a larger information maintenance period is conceivable if there is an age-related reduction of WM capacity.

Similarly, previous neuroimaging studies of long-term episodic memory retrieval have revealed a posterior to anterior shift of brain activation with aging, as exposed in the Posterior-Anterior Shift in Aging (PASA) hypothesis [24]. Furthermore, it has been suggested that it may be a common mechanism to compensate for memory demands exceeding an individual’s capacity [19]. Thus, this recruitment of anterior or frontal resources is supposed to occur at lower levels of load with aging to compensate for a decrease in posterior (more specific) processing resources. Nonetheless, to the best of our knowledge, this posterior to anterior shift in brain activity with aging and its interaction with memory load has not been studied with the ERP technique during WM recognition processes yet.

Consequently, the present exploratory study analyzed the ERP components during WM recognition stage of a DMS task with two levels of visual memory load and two maintenance period durations. Hence, this study aimed to explore the effects of aging on brain electrical activity during WM recognition; in particular, taking advantage of the ERP time resolution, the study aimed to assess whether there is a generalized age-related slowing of processing speed reflected in longer peak latencies of the ERP components. In addition, ERP components amplitude and brain electrical activity distribution and estimated sources were analyzed to investigate if there are age-related differences in the allocation of processing resources as well as whether these differences are dependent on WM demands.

## Materials and Methods

### Sample

The sample comprised 40 volunteers. All except three were right handed, as assessed by the Edinburgh Handedness Inventory [25]. All participants had normal or corrected-to-normal vision and reported no history of neurological or psychiatric disorders. None of the participants were taking psychotropic medication and they were instructed to abstain from consuming alcohol and caffeine the day prior to the experimental session. All participants gave their written informed consent before the experimental session in accordance with the Declaration of Helsinki, and the study protocol was approved by the ethical committee of the University of Santiago de Compostela (USC).

Participants were further divided in two groups (with 16 females each): 20 young adults (mean age = 23.85 ± 3.18 years) were recruited from the USC alumni, and 20 healthy old adults (mean age = 67.80 ± 7.69 years) were recruited from USC courses for older adults and from two different cultural associations where they participate in cognitively demanding activities (foreign language learning, informatics courses, etc.). The two groups obtained equivalent scores in the Spanish version of the Wechsler Adults Intelligence Scale vocabulary subtest [26] (young: 47.95 ± 5.22, old: 48.15 ± 8.68; t(31.41) = 0.89, p≤.930), albeit they differed significantly in time spent in formal education (young: 16.25 ± 1.25 years, elderly: 14.12 ± 3.93 years; t(18.79) = -2.160, p≤.044).

### Experimental protocol

**Fig 1:**
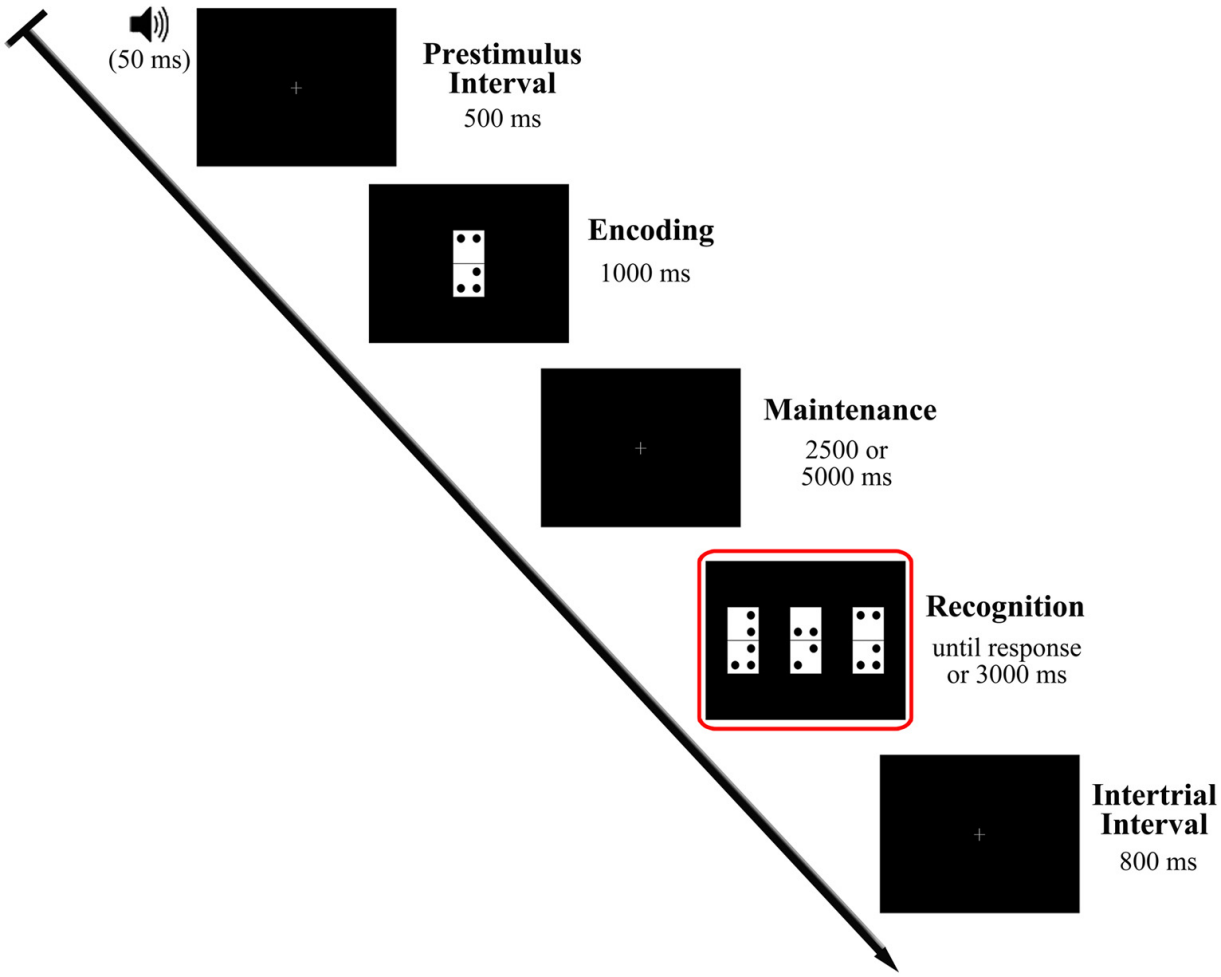
Diagram of the delayed match to sample task used in the study. The recognition stage, which was the focus of the analysis carried out in the present study, is represented inside the red square.

Participants performed the visual DMS task illustrated in Fig 1, which is described in detail elsewhere [22]. The task comprised 200 trials divided into two blocks with a 5 minute inter-block interval. Participants also received a short training on the task before EEG registration. Each trial began with a warning tone (1000 Hz pitch, 50 ms duration) indicating the start of a 500 ms prestimulus interval. A domino tile (sample stimulus), was then presented (encoding stage) and remained in the screen for 1000 ms. A blank screen delay of 2500 or 5000 ms (50% of probability of appearance) (maintenance period), was followed by presentation of three new dominoes (probe stimuli) (recognition stage). The probe stimuli were presented until the participant’s response or a maximum of 3000 ms, and the inter-trial interval was 800 ms. To minimize ocular artifacts, a fixation cross was placed in the center of the screen during the periods in which no stimulation was presented. Presentation of stimuli and recording of responses were controlled by Presentation^®^ software (Neurobehavioral Systems, Inc., Albany, CA, USA).

In the present study analyses were restricted to the probe stimulus presentation, during which participants were required to retrieve sample stimulus information, compare it with the new visual input and identify the previously presented domino tile. Each domino tile (8 cm high x 4 cm wide) consisted of two equally sized, vertically arranged white squares. Further, a single tile included a total of two to five black dots (1 cm diameter) located at eight possible positions (i.e. one per corner of each of its two squares), leaving a 1 cm gap between them and a 0.5 cm gap from the square boundaries. No more than three of these dots were placed in a single square (tile half). Each sample stimulus was presented on a black background in the center of a 19" monitor (refresh rate: 100 Hz) located at a distance of 1 m from the participant's eyes, so that it subtended a visual angle of 4.58° x 2.28°. As regards probe stimuli, the dominoes were presented simultaneously in the horizontal midline of the monitor (4 cm apart from each other). Only one was identical to the sample stimulus (target), and participants were asked to identify this as quickly and accurately as possible by pressing the button corresponding to its position on screen (left, center or right) from three response buttons arranged horizontally on a response device (Cedrus ^®^, model RB-530). The response button was counterbalanced across trials so that no more than three consecutive trials involved pressing the same button.

Memory load was manipulated between trials by changing the number of dots on the dominoes. The dominoes were grouped into two memory load conditions: a low load condition (LL) comprising dominoes with two or three dots, and a high load condition (HL) comprising dominoes with four or five dots. All dominoes in a trial pertained to the same memory load condition. The first task block consisted of 90 low memory load trials, while the second block consisted of 110 high memory load trials. The percentage of repeated dominoes was maintained constant in both blocks (20%). The HL block included more trials than the LL block to ensure a good signal-to-noise ratio, since a higher proportion of errors was expected for that block.

Maintenance period duration was also manipulated. Two conditions were generated: a short maintenance period (SMP) including trials with a 2.5 s delay between sample and probe stimuli, and a long maintenance period (LMP) comprising trials with a 5 s delay between the stimuli. Maintenance period duration was pseudo-randomly distributed across trials so that half of the trials in each block pertained to each condition, while avoiding more than five consecutive trials with the same maintenance period duration.

### EEG recording and signal processing

During the experimental session, participants sat in a comfortable armchair inside a noise and light attenuated Faraday chamber. Consistent with the methods used in previous studies [14–17], EEG activity was recorded through 51 active electrodes inserted in a cap and placed in the standard positions of the 10–10 system, with fronto-polar ground and nose tip reference. All electrode impedances were maintained below 8 kΩ. EOG activity was monitored by placing two electrodes in the outer canthi of both eyes (HEOG) and another two electrodes above and below the right eye (VEOG). The EEG signal was bandpass filtered (2^nd^ order phase-shift free Butterworth filter between 0.01 to 100 Hz), sampled at 500 Hz and digitally recorded for off-line analyses.

The recorded data were passed through a digital phase-shift free Butterworth filter with the high cut-off frequency at half power (-3 dB) set at 30 Hz (12 dB/octave roll-off) and with a low cut-off frequency at half power set at 0.1 Hz (12 dB/octave roll-off). A notch-filter centered at 50 Hz was also applied to avoid contamination of electrical line noise. Ocular and muscular artifacts were corrected using the Infomax algorithm in an Independent Component Analysis as implemented in Brain Vision Analyzer (v.2 Brain Products GmbH). Semi-automatic artifact rejection was also conducted (i.e. trials with voltage changes of ±125 μV were excluded). Data were segmented in epochs ranging from 200 ms prior to probe stimuli presentation to 1000 ms post-stimuli, and they were baseline corrected with the mean activity in the 200 ms prior to the probe stimuli onset. Only epochs corresponding to correctly answered trials were included in further analyses.

### Behavioral and electrophysiological data

The proportion of correct responses and reaction times (RTs) for the trials with correct responses were registered for each participant and experimental condition (LL-SMP, LL-LMP, HL-SMP and HL-LMP). Both measures were combined in the Inverse Efficiency (IE) score, which is calculated as the mean RT divided by the proportion of correct responses [27]. An increase in RT or decrease in the proportion of correct responses would thus result in an increase in the IE score; hence, higher IE scores reflect poorer performance than lower scores. IE can be considered as a “corrected reaction time” and its use avoids possible contamination of speed-accuracy tradeoffs or response criterion shifts [28]. The IE was calculated for each subject and each condition.

As regards electrophysiological data, separate averaged ERP waves to probe stimuli were obtained for each experimental condition (LL-SMP, LL-LMP, HL-SMP and HL-LMP). On the basis of the reports reviewed in the introduction section as well as visual inspection of the ERP waveform, the components of interest for the present study were: P1, N1, P2, N2, P300; and a Negative Slow Wave (NSW). Peak latency and baseline to peak amplitude of these ERP components were measured as follows: P1 was considered the maximum peak at O1, Oz and O2 between 85 and 145 ms post-stimuli; N1 was defined as the most negative peak between 150 and 210 ms after stimuli presentation at P9, P7, P8 and P10; P2 was measured as the largest positive peak at F3, Fz and F4 between 180 and 250 ms post-stimuli; N2 was considered the maximum negative peak at F3, Fz, F4, C3, Cz and C4 between 230 and 300 ms after probe stimuli onset and always as the negative deflection preceding the P300 component; P300 was identified as the maximum positive peak separately at P3, Pz and P4, and at F3, Fz and F4, between 300 and 500 ms post-stimuli. For each component, latency and amplitude data were averaged across the selected electrodes, so that the mean values for each component were included in the statistical analyses. As regards the NSW, the mean amplitudes between 700 and 850 ms post-stimuli and from 851 to 1000 ms post-stimuli were measured at Fz, Cz, Pz and Oz electrodes.

Table 1 includes the mean values and standard deviation of these ERP parameters for each group in each experimental condition.

**Table 1:** Parameters of ERP components.

		P1		N1		P2		N2		P3 Frontal		P3 Parietal		NSW 701–850 ms	NSW 851–1000 ms
		*Lat*	*Amp*	*Lat*	*Amp*	*Lat*	*Amp*	*Lat*	*Amp*	*Lat*	*Amp*	*Lat*	*Amp*	*Amp*	*Amp*
**Elderly**	**LL**	105.1 ± 15.9	3.7 ± 3.7	175.9 ± 15.8	-4.3 ± 2.9	201.9 ± 12.9	3.0 ±3.1	292.1 ± 15.6	-4.8 ± 2.5	414.4 ± 27.7	1.8 ± 1.7	417.0 ± 29.5	1.6 ± 3.2	-2.6 ± 3.1	-3.0 ± 3.5
	**HL**	105.2 ± 14.2	3.3 ± 2.8	173.7 ± 13.2	-3.8 ± 2.5	203.9 ± 15.4	2.9 ± 3.3	296.2 ± 15.6	-3.8 ± 2.5	404.0 ± 40.3	1.6 ± 1.7	419.2 ± 30.3	1.4 ± 3.0	-2.0 ± 2.8	-2.8 ± 2.6
	**SMP**	103.6 ± 15.3	3.8 ± 3.5	174.1 ± 14.1	-4.4 ± 2.6	201.5 ± 13.4	2.3 ± 3.7	288.5 ± 20.2	-4.6 ± 3.3	408.9 ± 39.7	1.6 ± 2.2	412.5 ± 38.0	1.1 ± 3.2	-2.9 ± 3.7	-3.5 ± 3.9
	**LMP**	106.8 ± 16.9	3.3 ± 3.3	175.5 ± 15.7	-3.8 ±3.4	204.2 ± 18.3	3.6 ± 2.9	299.8 ± 17.6	-4.0 ± 2.5	409.5 ± 38.8	1.7 ± 1.6	423.7 ± 28.4	1.8 ± 3.3	-1.8 ± 2.7	-2.4 ± 2.9
**Young**	**LL**	104.9 ± 17.7	4.5 ± 5.1	172.4 ± 15.7	-4.4 ± 2.5	198.4 ± 11.2	3.4 ± 3.4	271.2 ± 15.4	-4.5 ± 3.9	366.6 ± 34.8	1.1 ± 3.0	374.0 ± 32.1	3.5 ± 5.5	-4.6 ± 4.6	-5.5 ± 4.7
	**HL**	105.6 ± 16.8	4.7 ± 4.2	175.5 ± 13.3	-3.7 ± 2.6	199.1 ± 13.9	4.0 ± 2.8	269.8 ± 17.6	-3.1 ± 4.2	358.8 ± 36.1	1.7 ± 3.1	378.0 ± 37.9	2.9 ± 5.6	-4.1 ± 5.0	-5.1 ± 5.0
	**SMP**	106.6 ± 17.9	5.2 ± 4.8	175.7 ± 13.2	-3.0 ± 2.4	197.5 ± 11.4	3.5 ± 3.5	269.3 ± 17.2	-4.0 ± 4.3	357.8 ± 34.4	1.3 ± 3.4	372.0 ± 37.2	3.0 ± 5.8	-4.9 ± 5.3	-5.7 ± 5.0
	**LMP**	103.9 ± 16.6	4.0 ± 4.7	172.2 ± 15.4	-5.1 ± 2.9	200.0 ± 14.3	3.9 ± 2.9	271.6 ± 16.6	-3.5 ± 4.2	367.7 ± 43.7	1.5 ± 2.9	380.0 ± 44.4	3.4 ± 5.5	-3.8 ± 4.8	-4.9 ± 5.3

Latency (Lat), in milliseconds, and amplitude (Amp), in μV, for each group and memory load condition. Mean value ± standard deviation.

In addition, standardized low-resolution electromagnetic tomography (sLORETA) software [29] was used to estimate the cortical sources of the EEG activity recorded at the scalp. To that end, sLORETA calculates the smoothest 3D current density distribution that would explain the surface potentials based on a 3-shell spherical head model registered to the Talairach human brain atlas. This model is divided in 6430 voxels representing the cortical gray matter and the hippocampus with a 5 mm spatial resolution. This calculation was made for the mean brain electrical activity of each participant across trials pertaining to each memory load (LL and HL) as well as maintenance period duration (SMP and LMP) conditions and separately for each time window corresponding to the aforementioned ERP components. That is, between 85 and 145 ms post-stimuli (P1 time window), between 150 and 210 ms after stimuli presentation (N1 time window), between 180 and 250 ms post-stimuli (P2 time window), between 230 and 300 ms after probe stimuli (N2 time window), between 300 and 500 ms post-stimuli (P300 time window) and between 700 and 1000 ms post-stimuli (NSW time window).

### Statistical analysis

In this exploratory study, age-related effects on behavioral performance, as reflected in the IE scores for the visual DMS task, and also those of memory load and duration of the maintenance period were assessed by a mixed design analysis of variance (ANOVA), with the between subjects factor Age Group (young and elderly) and the within-subject factors Memory Load (LL and HL) and Maintenance Duration (SMP and LMP).

In order to explore age-related effects on the ERP parameters as well as the potential effects of memory load and duration of the maintenance period, mixed design ANOVAs were applied to the latency and amplitude of the ERP components P1, N1, P2, N2, with the between subjects factor Age Group (young and elderly) and the within subjects factors Memory Load (LL and HL) and Maintenance Duration (SMP and LMP). Two mixed design ANOVAs with the same factors were applied to the mean amplitude values of the NSW between 700 and 850 ms post-stimulus and from 851 to 1000 ms after stimulus onset.

For P300, which was measured at frontal and parietal sites, mixed design ANOVAs were applied to the amplitude and latency values, with the between subjects factor Age Group (young and elderly) and the within subjects factors Memory Load (LL and HL), Maintenance Duration (SMP and LMP) and Region (frontal and parietal).

In all the mixed ANOVAs performed in the present study statistical significance was set at p≤.05. Greenhouse-Geisser correction was applied whenever the sphericity assumption was violated, while Bonferroni adjustment was used to control for multiple comparisons.

Also, in sLORETA, the obtained current density distributions in source space were compared between age groups for each memory load as well as maintenance duration condition by running a nonparametric statistical mapping procedure [30]. Hence, for each single voxel, an independent sample log of ratio of averages (similar to log of F-ratio) test comparing amplitude estimates for the two groups at each defined time window was run. Then, 10000 randomizations with the statistical procedure applied in each run were conducted to determine a statistical threshold (p < 0.05) corrected for multiple comparisons.

## Results

### Behavioral performance

ANOVA of the IE scores revealed a significant main effect of the Age Group factor [F(1,38) = 65.43, p<0.001], as well as a significant main effect of the Memory Load factor [F(1,38) = 91.76, p<0.001].

**Fig 2:**
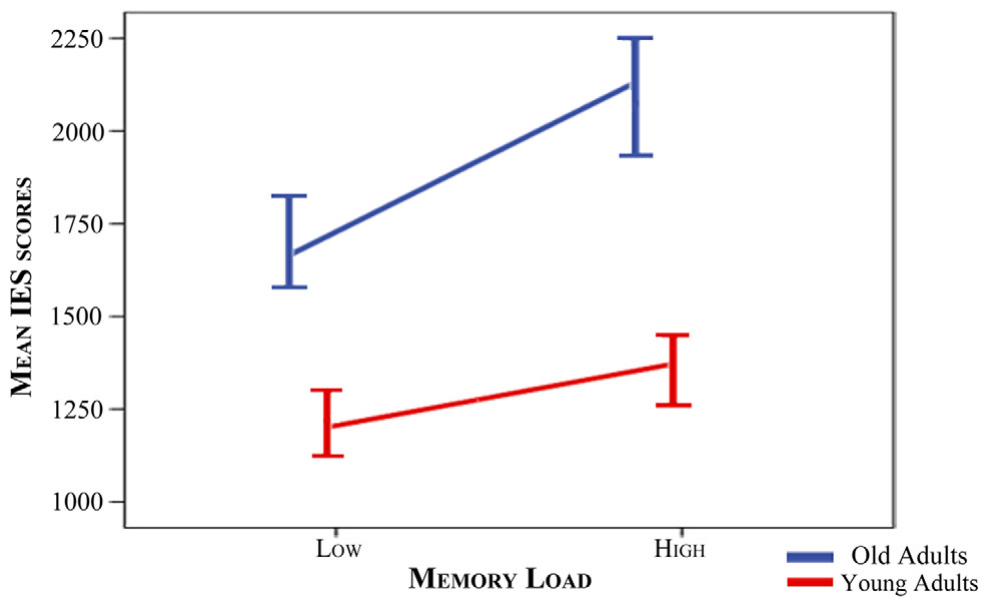
Increase in IE scores from low to high memory load conditions for each group. Segments depicting the slope of the increase in mean IE scores from the low load memory condition to the high load memory condition for each group (blue: old adults, red: young adults). Bars at the segment’s edges indicate standard errors (multiplied by 2).

The interaction between both factors was also statistically significant [F(1,38) = 19.73, p<0.001]. Thus, IE scores were longer (note that they are expressed in ms) for old than for young adults in both memory load conditions (p≤0.001), while high load conditions yielded significantly longer IE scores than low load conditions in both age groups (p≤0.001) (Table 2). To further explore this interaction, IE scores for the low load condition were subtracted from those of the high memory load for each participant. A t-test was used to compare both age groups. Significantly longer values were observed in the old than in the young group [t(27.05) = 4.44, p<0.001], indicating greater increases of IE scores with memory load in the older than in the young group (Fig 2).

**Table 2:** Behavioral data.

	Elderly						Young					
	Low Load			High Load			Low Load			High Load		
	IE	RT	Acc	IE	RT	Acc	IE	RT	Acc	IE	RT	Acc
Short Maintenance Duration	1694.6 ± 307.5	1450.6 ± 192.9	86.6 ± 8.4	2169.4 ± 352.1	1689.8 ± 163.5	79.3 ± 11.0	1137.1 ± 229.2	1061.6 ± 193.0	93.7 ± 4.2	1324.1 ± 234.1	1230.7 ± 203.7	93.2 ± 3.5
Long Maintenance Duration	1727.4 ± 340.3	1530.1 ± 208.8	89.9 ± 8.9	2184.0 ± 491.7	1668.2 ± 175.6	78.8 ± 13.1	1117.7 ± 232.0	1079.8 ± 208.7	96.9 ± 3.2	1272.0 ± 257.6	1187.8 ± 220.3	93.7 ± 3.7

Inverse Efficiency (IE) scores and reaction times (RT), expressed in ms, and accuracy rates (Acc), expressed as percentages, for each group in each memory load and maintenance duration condition. Mean value ± standard deviation.

No significant main effect or interaction was observed for the factor Maintenance Duration.

### Brain electrical activity

#### ERP Latencies

As regards latencies of the ERP components, ANOVAs revealed significant main effects for the factor Age Group on N2 and P300 [F(1,38) = 25.52, p<0.001 and F(1,38) = 24.16, p<0.001, respectively]. In both cases, latencies were significantly longer in old than in young adults.

**Fig 3:**
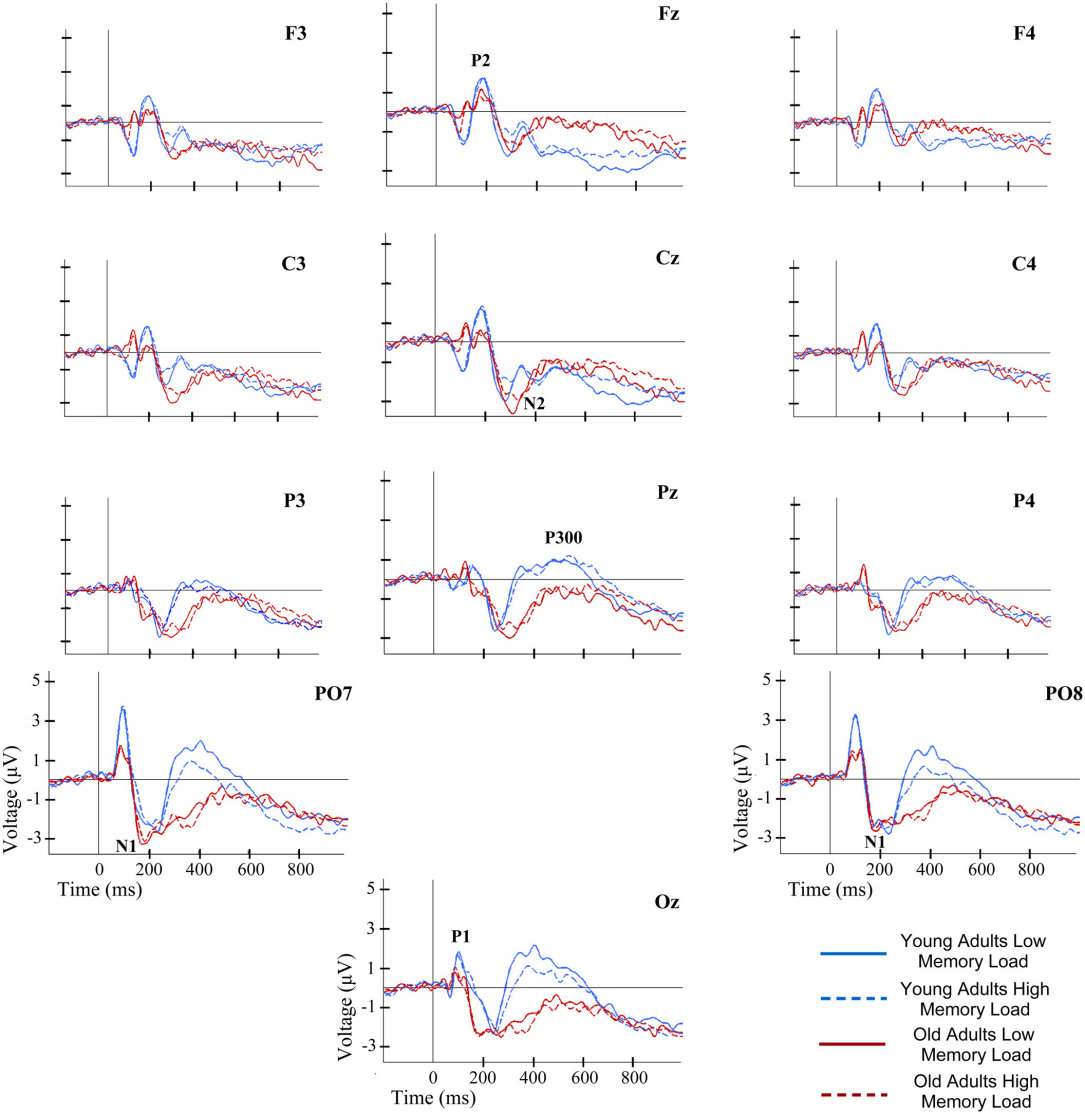
ERP waveforms at several electrode locations during recognition of information in WM. ERP waveforms for each group and memory load condition.

No significant main effects of Memory Load were observed. However, a significant interaction between the factors Memory Load and Region was observed for P300 [F(1,38) = 6.04, p≤0.019]; latencies were significantly longer in HL than in LL conditions at frontal electrodes (p≤.039) (Fig 3).

**Fig 4:**
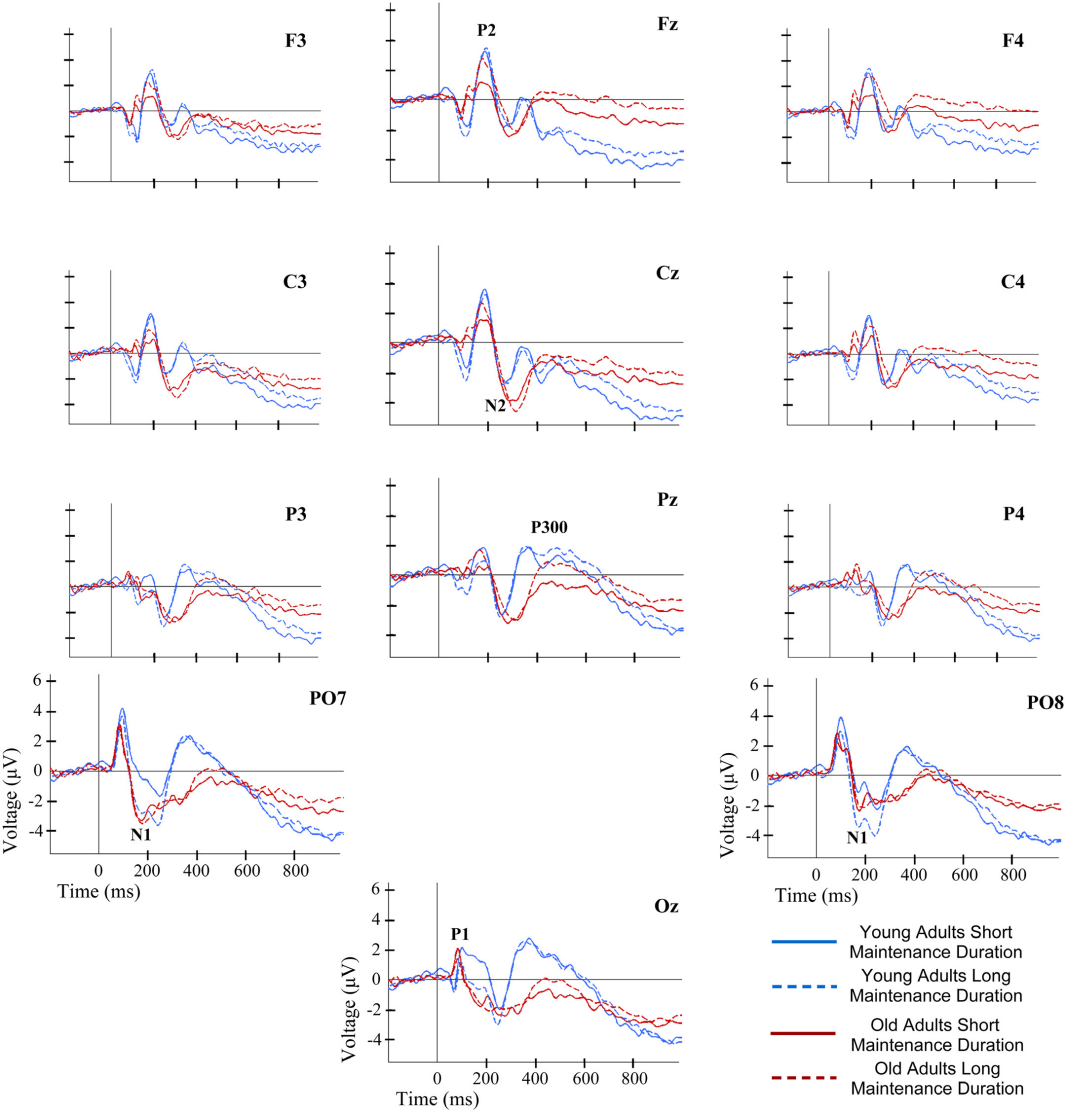
ERP waveforms at several electrode locations during recognition of information in WM. ERP waveforms for each group and maintenance duration condition.

Significant main effects involving factor Maintenance Duration were observed for N2 [F(1,38) = 4.53, p≤0.040]. N2 latencies were significantly longer in LMP than in SMP conditions (Fig 4).

#### ERP Amplitudes

No main effects involving factor Age Group were found to be statistically significant in the ANOVAs applied to the amplitudes of ERP components. Nevertheless, a significant interaction between Age Group and Maintenance Duration was found for N1 amplitude [F(1,38) = 11.07, p≤0.002], with significantly larger amplitudes in LMP than in SMP trials only for the young adults (p≤0.001). A significant interaction between Age Group, Memory Load and Region was observed for P300 [F(1,38) = 7.08, p≤0.011]. Thus, in low load trials, the P300 amplitude was larger in parietal than in frontal regions only in the young adults (p≤.021) (Fig 3).

Significant main effects of factor Memory Load were found for N1 amplitude and for the NSW mean amplitude in the time window between 700 and 850 ms post-stimuli [F(1,38) = 6.79, p≤0.013 and F(1,38) = 5.14, p≤0.029, respectively]. In both effects, the amplitudes were significantly lower in HL than in LL trials (Fig 3).

As regards the Maintenance Duration factor, significant main effects were observed for P1 [F(1,38) = 5.64, p≤0.023] and P2 amplitudes [F(1,38) = 6.01, p≤0.019], as well as NSW mean amplitude in the time window between 700 and 850 ms post-stimuli [F(1,38) = 4.12, p≤0.049]. While the amplitudes of P1 and the NSW were significantly lower in LMP than in SMP conditions, the P2 amplitude was significantly larger in LMP than in SMP conditions (Fig 4).

#### Brain activation

**Fig 5:**
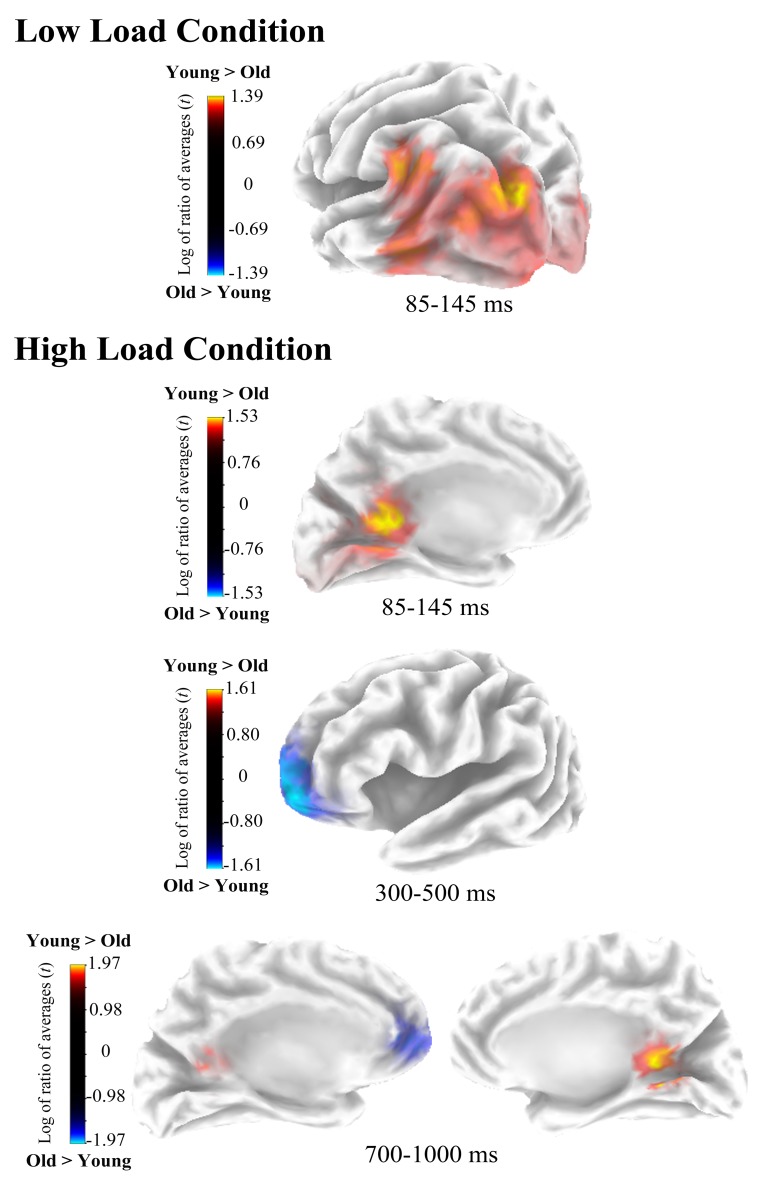
Estimated sources of activation differences between age groups. On top, cluster of voxels showing age-group differences in the low load condition, and on the bottom age group differences in brain electrical activity at source space in the high load condition.

In the low load condition, sLORETA analyses revealed that brain activity significantly differed between groups at P1 time window (p≤0.012). In this period (85–145 ms) activity was higher for young than for old participants in the left Cuneus (Brodmann Area—BA- 19) and midline posterior cingulate (BA 30) (Fig 5). Coordinates of the voxels showing maximal difference between groups, *t* values and the associated p values are shown in Table 3.

**Table 3:** Voxels showing maximal activation differences between age groups.

Coordinates			Brain region	Period	Statistics		
X	Y	Z			*t* value	Assoc. p	
Low Load condition							
-25	-86	37	Cuneus (BA 19)	85–145 ms	1.39	<0.05	Y>O
0	-48	16	Posterior Cingulate (BA 30)	85–145 ms	1.37	<0.05	Y>O
High Load condition							
0	-48	16	Posterior Cingulate (BA 30)	85–145 ms	1.51	<0.01	Y>O
-25	63	-7	Superior Frontal Gyrus (BA 10)	300–500 ms	-1.61	<0.05	O>Y
5	-48	12	Posterior Cingulate (BA 29)	700–1000 ms	1.97	<0.01	Y>O
-10	53	-3	Superior Frontal Gyrus (BA 10)	700-1000ms	-1.83	<0.01	O>Y

In addition, in high load conditions, significant differences in brain activation between the two age groups were found at P1 (p≤0.002), P300 (p≤0.030) and NSW (p≤0.003) time windows. During the first window (85–145 ms) activity was significantly higher for young than for old adults in the midline posterior cingulate (BA 30). In contrast, in P300 time window (300–500 ms) activity was significantly higher for old than for young participants in the left superior frontal gyrus (BA 10). Finally, in the NSW time window (700–1000 ms) activity was significantly higher for young than for old participants in the right posterior cingulate (BA 29) and *vice versa* for the left superior frontal gyrus (BA 10) (Fig 5).

No differences between groups in brain activation were observed for the short or the long maintenance period conditions in sLORETA analyses.

## Discussion

### Task performance

Performance of the DMS task was poorer in old than in young adults. This is consistent with previous findings indicating an age-related WM decline in the execution of different cognitive tasks [1]. In addition, high memory load also yielded a decrease in task performance; this is in accordance with previous findings of a detrimental effect of memory load on task performance [6,7,22].

Interestingly, the detrimental effect of memory load was more marked in old than in young adults. This effect was not observed in previous studies with DMS tasks that used visually presented digits [14,17]. This significant interaction between memory load and age effects is consistent with the findings of a previous study with a DMS task that used abstract shapes as stimuli [31]. Consequently, it seems that at a behavioral level, older adults are more sensitive to memory load than young adults when performing tasks with complex visual stimuli.

Maintenance duration, on the other hand, had no effect on task performance, which contrasts with previous findings [31] of significant effects of maintenance duration on the execution of young and old adults in visuospatial DMS tasks with shorter retention intervals (i.e. 500 and 2500 ms) than those used in the present study. This difference may explain the contradictory findings. Indeed, a previous study has suggested differences in the strategy used by participants to maintain spatial information of visually presented stimulus between very short intervals (500 ms), in which participants employed a strictly visual/perceptual strategy, and long intervals (2.500 and 5000 ms), in which participants reported a combination of visual and verbal strategies [32]. Therefore, the present results suggest that using relatively long maintenance periods (i.e. 2500 and 5000 ms) in visuospatial DMS tasks, the behavioral performance of old and young adults might not be affected by the amount of time that information should be held in mind.

### Brain electrical activity during WM recognition

#### ERP latencies

Age-related differences in brain electrical activity timing began at around 250 ms after presentation of probe stimuli and were reflected as longer N2 and P300 latencies in old than in young adults.

In contrast with the age-related modulation of N1 latency during WM recognition reported in previous studies [14,15], no differences in N1 latency between age groups were observed in the present study. This component has been associated with perceptual processing of complex visual inputs [33,34], and its latency has been shown to increase with increasing processing effort and when local rather than global features of the stimuli are task relevant [35,36]. In contrast with the present study, in which the configuration of dots inside the domino was relevant, in those previous studies showing age-related differences in N1 latency only the identity of digits [14] or the position of a single dot [15] were relevant. Therefore, it could be the case that in the present study there was a greater variability on N1 peak latencies due to greater relevance of local aspects of the configuration of the visual stimuli and to a higher degree of processing effort than in previous studies. This variability could have obscured potential age-related differences in N1 latency. Consequently, while the present findings may indicate preserved perceptual processing speed for complex visual stimuli during WM recognition in old adults, future studies that experimentally manipulate these factors have to be performed to reach firm conclusions.

The N2 component has been related to initial evaluation of the stimulus [37], which includes evaluation of the probe stimuli and comparison of each domino with the memory template of the sample stimulus during WM recognition [38]. Similarly, P300 latency is considered an indicator of the amount of time required for completion of stimulus evaluation and classification processes [39,40]. Although some studies did not find age-related differences in N2 latency [16,17], longer P300 latencies with aging are consistent with previous findings during WM recognition in DMS tasks [14,16,17], as well as other kind of tasks [for review see 2]. Thus, the present results may extend previous findings as they reveal that age-related slowing of processing speed during WM recognition affects probe stimuli evaluation and classification processes.

Additionally, memory load affected P300 latency. Longer P300 latencies in high than in low memory load trials were found, which is consistent with previous findings in P300 research [40,41]. Hence, the present finding may indicate that, during WM recognition, evaluation and classification of the probe stimuli -based on the memory template of the sample stimulus- takes longer in the high than in the low memory load condition, independently of age.

With regard to maintenance duration effects, longer N2 latencies were found in trials with long than in trials with short durations, in both age groups. Thus, after long maintenance periods, initial evaluation of probe stimuli and comparison with the memory template of the sample stimulus is probably delayed relative to short maintenance periods. Tentatively, it could be argued that this effect is probably related to the weakening of the memory traces due to the mere passage of time during the maintenance period, as suggested from time-based decay theories [42].

In summary, the present results of the ERP components latency exploratory analysis add evidence indicating that there is an age-related slowing of processing speed. However, such slowing may be specific for stimulus evaluation and classification processes. Also, irrespective of age, it seems that memory demand slowed stimulus classification and stimulus evaluation when modulated by memory load or by maintenance duration, respectively.

#### ERP amplitudes

Although there were no main effects of age group in the present exploratory study, age-related effects interacted with those of maintenance duration on N1 and those of memory load on P300.

The N1 amplitudes were only larger after long than after short maintenance periods in the young participants. Lower N1 amplitude during WM recognition under high than under low cognitive demands has been interpreted as a sign of a reduction in the amount of processing resources allocated to perceptual processing, probably due to a greater distribution of these resources between perceptual processing of new visual inputs and maintenance of previously seen visual stimuli [20]. Accordingly, in the present study it seems that young participants were able to allocate more processing resources to perceptual processing after long than after short maintenance periods, as the distribution of processing resources between maintenance and perceptual processes is lower in the former than the in latter condition. Interestingly, no such differences were observed in old adults.

The P300 amplitude was larger at parietal than at frontal sites in the young subjects during the low load condition, while no inter-electrode differences were observed in the older group. A topographic distribution with parietal maximum has been consistently reported in P300 research in healthy young adults [40], as well as a more homogeneous topographic distribution of this component amplitude in healthy old adults [43–45]. Such homogeneous distribution due to the loss of a clear parietal maximum for P300 amplitude has been considered an indicator of maintained or increased frontal activity with aging. Such changes are consistent with the proposed posterior to anterior shift with aging (PASA) hypothesis [24]. Thus, the present findings support the PASA hypothesis regarding WM recognition processes.

Furthermore, the results revealed that young adults showed under high memory load a more uniform P300 amplitude distribution -without a significant parietal maximum- than under low load conditions. On the other hand, old adults presented such homogenous distribution in both memory load conditions. Together with behavioral results, this may indicate that old adults were more sensitive to memory load than their younger counterparts, which is well in line with hypothesis pointing to a reduced capacity of WM with advancing age.

Independent of the age of participants, memory load affects the amplitude of N1 and the mean amplitude of the first period of the NSW. The amplitudes were smaller in high than in low memory load trials in both cases. In the DMS task, recognition of the domino tiles in the high load condition probably requires the distribution of processing resources to more mental operations as well as greater effort than in the low load condition. Therefore, when there is higher number of dots in the domino tile, it seems that more effort is allocated to the processing of its spatial configuration as well as for carrying out post-categorization and response preparation processes. This may yield a reduction in N1 and NSW amplitudes, as these components presumably reflect each of these processes, respectively [21,22].

Maintenance period duration modulated P1 and P2 amplitudes, and also the NSW mean amplitude in its first interval for both age groups. The amplitudes of P1 and NSW were smaller after long than after short maintenance periods, while the amplitude of P2 was larger. Although it is difficult to interpret the effects on brain electrical activity found for maintenance duration in the present study, it seems that maintenance duration modulates cognitive demands, as previously suggested [8]. The effects of maintenance duration were independent of the age of participants. Therefore, considered together with behavioral results, the present pattern of results may indicate that old and young adults are similarly affected by maintenance duration at both behavioral and electrophysiological levels. This contrasts with the suggested age-related reduction in WM capacity. Accordingly, future work is warranted to further our understanding of the impact of maintenance duration on the brain activity underlying WM recognition processes.

#### Brain Activation

Age-related differences in brain activation in both memory load conditions were observed. Posterior cingulate cortex (BA 30 & 29) showed lower activation for old than for young adults between 85 and 145 ms post-stimulus in both memory load conditions and between 700 and 1000 ms only in the high load condition. In contrast, in this latter interval as well as between 300 and 500 ms (in the P300 time window) older adults showed higher activation than young adults in left superior frontal gyrus. Taken together these results are in accordance with the PASA hypothesis. Therefore, it seems that during WM recognition old adults have a greater reliance in frontal relative to posterior processing resources than young adults.

## Conclusion

In summary, results from the exploratory analysis performed in the present study indicate that: behavioral data shows an age-related decline in performance of a visuospatial WM task. Also, they add evidence in favor of a greater sensitivity to WM load in old relative to young adults. The electrophysiological data showed a specific age-related slowing in the evaluation and classification of the probe stimulus (which triggers the recognition process), but not in its perceptual processing. The data also indicated a more homogeneous scalp distribution of P300 in old than in young adults. This, together with the age-related reduced posterior cingulate cortex activation and increased superior frontal gyrus activation, support an age-related posterior to anterior shift in the recruitment of neural resources during WM recognition.
